# Computational identification of differentially-expressed genes as suggested novel COVID-19 biomarkers: A bioinformatics analysis of expression profiles

**DOI:** 10.1016/j.csbj.2023.06.007

**Published:** 2023-06-12

**Authors:** Valentina Di Salvatore, Elena Crispino, Avisa Maleki, Giulia Nicotra, Giulia Russo, Francesco Pappalardo

**Affiliations:** a Department of Drug and Health Sciences, University of Catania, Catania, Italy; b Department of Biomedical and Biotechnological Sciences, University of Catania, Catania, Italy; c Department of Mathematics and Computer Science, University of Catania, Catania, Italy; d Mimesis SRL, Catania, Italy

**Keywords:** SARS-CoV-2, COVID-19, RNA-Sequencing, Biomarker, Differential expression analysis, Gene set enrichment analysis

## Abstract

COVID-19 was declared a pandemic in March 2020, and since then, it has not stopped spreading like wildfire in almost every corner of the world, despite the many efforts made to stem its spread. SARS-CoV-2 has one of the biggest genomes among RNA viruses and presents unique characteristics that differentiate it from other coronaviruses, making it even more challenging to find a cure or vaccine that is efficient enough. This work aims, using RNA sequencing (RNA-Seq) data, to evaluate whether the expression of specific human genes in the host can vary in different grades of disease severity and to determine the molecular origins of the differences in response to SARS-CoV-2 infection in different patients. In addition to quantifying gene expression, data coming from RNA-Seq allow for the discovery of new transcripts, the identification of alternative splicing events, the detection of allele-specific expression, and the detection of post-transcriptional alterations. For this reason, we performed differential expression analysis on different expression profiles of COVID-19 patients, using RNA-Seq data coming from NCBI public repository, and we obtained the lists of all differentially expressed genes (DEGs) emerging from 7 experimental conditions. We performed a Gene Set Enrichment Analysis (GSEA) on these genes to find possible correlations between DEGs and known disease phenotypes. We mainly focused on DEGs coming out from the analysis of the contrasts involving severe conditions to infer any possible relation between a worsening of the clinical picture and an over-representation of specific genes. Based on the obtained results, this study indicates a small group of genes that result up-regulated in the severe form of the disease. EXOSC5, MESD, REXO2, and TRMT2A genes are not differentially expressed or not present in the other conditions, being for that reason, good biomarkers candidates for the severe form of COVID-19 disease. The use of specific over-expressed genes, whether up-regulated or down-regulated, which have an individual role in each different condition of COVID-19 as a biomarker, can assist in early diagnosis.

## Graphical Abstract

**Figure:**
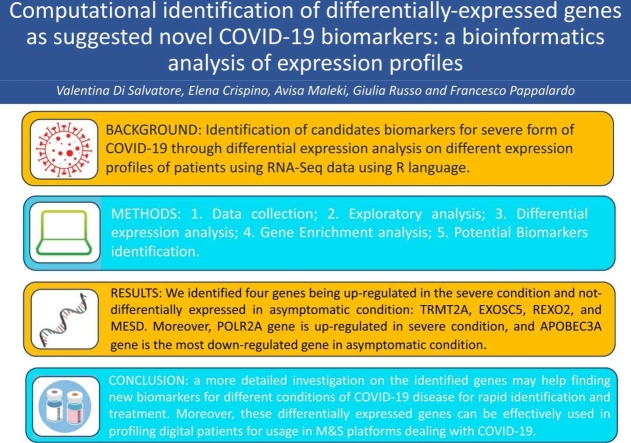

## 1 Introduction

SARS-CoV-2 is a single-stranded ribonucleic acid beta coronavirus with an envelope. Coronaviruses have one of the biggest genomes of any RNA virus, measuring between 27 and 32 kb. The membrane and envelope proteins are required for virus assembly, while the spike protein is required for virus entrance and recognition by the host cell (3). The major process through which the virus enters host cells is receptor-mediated endocytosis. SARS-CoV-2 infects lung AT2 alveolar respiratory tract epithelial cells using angiotensin-converting enzyme 2 (ACE2), a cell-surface receptor found in the kidney, blood vessels, and heart [1], [2].

SARS-CoV-2 is the eighth coronavirus to infect humans, and it can cause a severe acute respiratory syndrome, especially in elderly people and people with comorbidities and immunocompromised states [2]. Respiratory droplets and aerosols, direct contact with mucosal membranes, and maybe the fecal-oral pathway are all ways to spread this highly contagious virus [3]. Asymptomatic carriers usually facilitate human-to-human transmission during incubation, lasting 5–7 and 28 days [3]. COVID-19 symptoms affect the respiratory tract in various ways, ranging from mild to severe. Because the respiratory system and the lungs are the most infected locations with COVID-19, pulmonary signs and symptoms are more numerous, apparent, and better documented. Patients infected with SARS-CoV-2 can progress their clinical status from fever, cough, ageusia and anosmia, sore throat, breathlessness, fatigue, or malaise to pneumonia, acute respiratory distress syndrome (ARDS), and multi-organ dysfunction illness [4], [5]. Other organs and systems, including the cardiovascular, liver, kidneys, gastrointestinal, and central nervous systems, are also affected with varying frequency and severity [6]. Patients with advanced age and comorbidities like obesity, hypertension, or diabetes mellitus may have a higher risk of severe disease and mortality.

The different symptoms and, therefore, the different conditions of subjects with COVID-19 may be related to the activation of some genes rather than others, making the genes themselves potential biomarkers for the severity of the disease. For this reason, we decided to investigate COVID-19 disease from a genetic point of view, using RNA sequencing data from a public repository. Using Bioconductor/R programming language, we performed differential expression analysis on three datasets coming from the NCBI GEO dataset repository. As a result, we obtained the complete set of differentially expressed genes (DEGs) coming from different contrasts specifically designed to probe the changes in expression levels of all genes involved in the progression of COVID-19 through the various stages of the disease. To go deeper into genetic mechanisms beyond COVID-19 evolution, we also performed Gene Set Enrichment Analysis (GSEA) on the DEGs list produced by differential expression analysis to identify all those biological processes and pathways in which these genes are enriched.

Since the onset of COVID-19 disease spread, scientists have put efforts into designing, discovering, and finding new drugs and compounds that are effective against the disease, as well as new methods able to define biomarkers involved in the pathogenesis of SARS-CoV-2 infection. Different methodologies concerning Artificial Intelligence (AI) and bioinformatic tools have been applied in the study of COVID-19 disease to screen approved pharmaceuticals against SARS-CoV2 proteins, thus finding medications for repurposing and determining whether the identified compounds may also influence human proteins whose expression in the lung changes after SARS-CoV2 infection [7]. AI has long been lauded as a possible means of assisting in identifying diseases and supporting clinicians in making correct diagnoses more quickly [8]. Its two main branches are machine learning (ML), which refers to the intelligence demonstrated by computers, and deep learning (DL), which is an enhanced and scalable ML extension that strengthens and facilitates the application of learning algorithms [9]. In COVID-19, deep learning extensively builds complex applications using massive datasets. One of the most widely used DL techniques is the convolutional neural network (CNN) [10], which, in the current era of DL-based approaches, has significantly increased its popularity in both general and medical image-based apps [11].

RNA-sequencing (RNA-Seq) methodology has also been widely used in the COVID-19 disease field [12]. One of the primary goals of COVID-19 single cell RNA-Seq research has been uncovering the genetic and cellular factors that drive COVID-19 disease severity. Finding upregulated and downregulated genes in single cell RNA-Seq data between COVID-19 patients and healthy controls, as well as between patients of varying severity, is one of the most frequent approaches [13]. Additionally, RNA-Seq has proven to be extremely sensitive and accurate at measuring cell proportions, making it easier to identify cells with abnormal proportions [14].

Researchers are developing drugs for COVID-19 treatment using various strategies. Among them, drug repurposing has successfully treated SARS-CoV-2 infection and bioinformatic methods and approaches have been demonstrated to be powerful tools in this context. Specifically, computational molecular modeling techniques have been employed to assess the potential of selected nucleoside analogs as prospective therapeutics against one of the Omicron subvariant [15], [16]. Inhibiting the COVID-19 polymerase has effectively halted the virus replication. In addition, two antioxidant polyphenolic compounds, CoViTris2020 and ChloViD2020, based on computational docking, showed very high inhibitory binding affinities with most of the docked SARS-CoV-2/human proteins [17], [18]. Ensitrelvir (S-217622) has also been discovered as a potent anti-coronavirus agent. This chemical was primarily derived from computational approaches employed in drug design and optimization [19]. These advancements highlight the potential of repurposing existing drugs for effective COVID-19 treatments and address the challenges posed by new viral variants [20].

According to the central dogma of molecular biology, which Crick first stated to explain the flow of genetic information in a biological system, all the information of a cell is contained in genes as DNA, then transcribed into RNA, and then translated into proteins [21], [22]. Thus, the transcription into RNA defines the cell's identity and controls its biological functions. RNA molecules are crucial for analysing the different parts of the genome and comprehending which roles some genes might play in certain diseases [23]. The RNA molecules are collectively known as the transcriptome, and the genome-wide quantification of gene expression is known as transcriptomics. The initial technologies used for transcriptomics studies were hybridization-based microarrays [24]. Transcriptomics has been revolutionized thanks to high-throughput next-generation sequencing (NGS), which allows RNA analysis using complementary DNA (cDNA) sequencing [25]. RNA-Seq is a technique that makes use of high-throughput next-generation sequencing (NGS) to analyse the transcriptome, and it gives significantly much more coverage and better resolution of the transcriptome’s dynamic nature than the earlier Sanger’s methods based on sequencing and microarray [26], [27]. RNA-Seq can show which genes encoded in the DNA are switched on or off and the extent to which they are turned on or off [28]. In addition to quantifying gene expression, data coming from RNA-Seq allow for the discovery of new transcripts, the identification of alternative splicing events (which result in the production of several transcripts from a single gene sequence), the detection of allele-specific expression, and the detection of post-transcriptional alterations. The usual steps of an RNA-Seq experiment consist of RNA extraction and isolation, conversion of RNA into complementary DNA (cDNA), cDNA library creation, and use of an NGS platform to sequence it [29]. The reasons why RNA-Seq is considered superior to microarrays are several, including its ability to detect RNA from organisms with previously unknown genomic sequences, its low background signal, and its ability to have more quantifiable data and to avoid problems that microarrays have in detecting remarkably high or exceptionally low transcription levels. Data coming from RNA sequencing can provide remarkable information about the human genome and its functional protein expression, leading to a deeper comprehension of cell biology and the discovery of specific changes that could indicate a particular disease.

The immune system's ability to fight against foreign pathogens depends on the strict control of immune gene expression. The control of RNA is a crucial stage in gene expression, and RNAs can be divided into two groups: those that code for proteins and those that do not, also called non-coding RNAs (ncRNAs) [30]. NcRNAs can be subclassified, according to their relative size, into short ncRNAs and long ncRNAs. MicroRNAs (miRNAs) are small ncRNAs that regulate human gene expression at the post-transcriptional level and which also play key roles in development, cancer, viral infections, and antiviral immune responses, among other physiological and pathological processes [31]. Viruses can globally interfere with the miRNA pathway: they may interact with the miRNA machinery of their hosts, thus increasing or suppressing the expression of specific miRNAs, and several viruses can encode their own viral miRNAs [31].

In SARS-CoV-2 infection, several studies have shown that ncRNAs and miRNAs may impact virus replication and the host immune response [32], [33]. For example, some miRNAs have been shown to target the viral genome or key host factors involved in viral replication, such as Neuropilin-1, a transmembrane glycoprotein that has been shown to be implied in the cellular internalization of the SARS-CoV-2 [34]. Moreover, many studies have been aimed at understanding whether miRNAs could be involved in the pathogenesis of thromboembolic complications of COVID-19 disease [35], as well as in the pathogenic mechanisms of chronic pain-like symptoms in patients with long COVID [36]. The altered long ncRNA expression has also been studied in COVID-19 patients and it was found to may be positively correlated with the severity of the disease [30], [37]. Furthermore, some ncRNAs and miRNAs have been proposed as potential therapeutic targets or diagnostic biomarkers for COVID-19 [38], [39].

One of the most critical steps in characterizing transcriptomic data is the identification of biomarkers within a set of differentially expressed genes from differential expression analysis conducted on RNA-Seq data [40]. When a gene is significantly up- or down-regulated in specific biological conditions, it can be further investigated to understand whether it can be considered a biomarker for a particular disease. However, even today, the identification of potential biomarkers on data coming from RNA-Seq analysis remains a challenge for many researchers, although, compared to traditional microarray analysis methods, many steps have been taken forward. This is mainly due to the fact that biomarkers identification techniques developed for normal data distributions are not applicable to RNA-Seq data, which are usually modeled from non-negative or Poisson binomial distributions [41]. In this study, we focused on the most differentially expressed genes across all conditions, taking into account both log2 FC and p-values, and then selected and investigated only those groups of genes showing dramatic changes in their expression level, going for example, from overexpressed in one condition to not expressed at all in another one.

The goal of this work is the identification of differentially expressed genes in subjects with different conditions of COVID-19 disease in order to understand the role of the genes themselves as well as to identify some possible biomarkers for each disease condition. In particular, we focused on genes that were up-regulated in patients with severe COVID-19 conditions, in comparison to other groups. Moreover, we focused on the analysis of the most overexpressed genes, both up- and down-regulated, in the asymptomatic and severe COVID-19 conditions, intending to understand how the differential expression of certain genes may affect the different severity degrees of the symptoms.

## 2 Material and methods

The RNA-sequencing analysis aims at the identification of transcripts and the quantification of gene expression for each specific organism under examination and finds large application within life science research, far beyond the field of genomics alone [42]. Even if there is no optimal pipeline for each of the possible applications of this technique, a typical RNA-seq workflow includes several steps:

1. Preliminary analysis, including
  a. Experimental design (choice of the library type, sequencing depth and appropriate number of replicates)
  b. Sequencing design
  c. Quality control (obtaining raw reads, read alignment and quantification)
2. Core analysis, including:
  a. Transcriptome profiling
  b. Differential gene expression
  c. Functional profiling
3. Advanced analysis, including:
  a. Visualization
  b. Data integration
  c. Results interpretation

R programming language (http://www.bioconductor.org/) offers a large variety of tools specifically designed for the analysis of data coming from RNA-sequencing. Each of these tools is applied to one specific step of the general RNA-seq analysis workflow, which usually includes a preliminary data quality control phase followed by the differential expression analysis and gene set testing [43].

Specifically, the methodology applied in this work includes the following steps:

1. Data collection
2. Exploratory analysis
3. Differential expression analysis
4. Gene Enrichment analysis
5. Potential Biomarkers identification

Each step is described in detail in the next paragraphs, and the results for each step are shown in the next chapter.

### 2.1 Data collection

Gene expression profile data have been downloaded from NCBI Gene Expression Omnibus (GEO) website (https://www.ncbi.nlm.nih.gov/geo/). We selected three datasets related to SARS-CoV-2, GSE166424, GSE178824 and GSE179448, for a total of 124 samples coming from GEO datasets and a total of 35,413 genes across all samples. We used “SARS-CoV-2″, “Human” and “RNA-Seq” as keywords to perform the search on the GEO database.

More specifically, dataset GSE166424 includes RNA samples extracted from whole blood collected from 30 asymptomatic patients from the Singapore cohort during active infection (PCR-positive). Asymptomatic patients with COVID-19 in this cohort study were primarily youth from Bangladesh, the Philippines and Burma. Dataset GSE178824 includes RNA samples collected from COVID-19 patients with different levels of severity and healthy controls. This study included 24 severe, 5 asymptomatic, 26 convalescent COVID-19 patients and 15 healthy controls. All patients tested positive for SARS-CoV-2 by PCR. Healthy controls were negative either by PCR or for antibodies to SARS-Cov-2. All severe patients were hospitalized in the intensive care unit for treatment because of hypoxia and respiratory distress or complications from pre-existing comorbidities. Among them, ten patients who died from COVID-19 complications underwent autopsy and samples were collected at the LSU Health Science Center Pathology Department. Finally, dataset GSE179448 includes samples coming from 84 COVID-19 patients and healthy control. In particular, COVID-19 patients (n = 57) were classified into: mild (outpatients); severe (hospitalized), more than half of whom were in intensive care (ICU), mostly sampled during the cytokine storm period, and recovered, virus-negative convalescents. We chose these three datasets for some specific reasons: first, they showed characteristics that best suited the type of study we were conducting, unlike many others that we had to discard. Moreover, the number of samples thus obtained was considered sufficient also from the point of view of statistical significance, so there was no need to add more data.

KEGG and GO (both biological process, molecular function and cellular component) hallmarks have been downloaded from the Molecular Signature Database (MSigDB, http://www.gsea-msigdb.org/gsea/msigdb/) and then used to run Gene Set Enrichment Analysis (GSEA) R script.

### 2.2 Exploratory analysis

Exploratory analysis is a fundamental, preliminary step in data analysis aimed at investigating the quality of data under examination and giving a general overview of their main features even before starting the actual analysis. During exploratory analysis, data are modeled, transformed, and prepared for the next steps of analysis, and the results of this initial stage are usually shown in a visual and intuitive way by plotting various explanatory graphs. To perform exploratory analysis, we used *ggplot* combined with *DESeq2* and *EdgeR* R packages. R (https://www.r-project.org) is a programming language specific for statistical computing and graphics. It is totally free and runs on different UNIX, Windows and MacOS environments. R allows the user to download and install different packages, specifically designed for particular tasks, from CRAN (https://cran.r-project.org) and Bioconductor (https://www.bioconductor.org) repositories.

### 2.3 Differential expression analysis

Differential expression analysis is a process consisting in performing a series of statistical analyses on normalized read counts data in order to highlight any quantitative changes in expression value levels between different experimental conditions. In this work, we focused on the overexpressed genes coming out from the following comparisons between experimental groups:

1. "Control_Healthy" vs "Recovered"
2. "Control_Healthy" vs "Asymptomatic”
3. "Control_Healthy" vs "Mild"
4. "Control_Healthy" vs "Moderate"
5. "Control_Healthy" vs "Moderate to severe"
6. "Control_Healthy" vs "Severe"
7. "Asymptomatic " vs "Severe"

To perform differential expression analysis, we used *DESeq2* R package (http://www.bioconductor.org/packages/release/bioc/html/DESeq2.html.). Among all the specific tools for the analysis of differential expression of RNA-Seq data, *DESeq2* has been selected for some of the characteristics that make it particularly suitable for the purposes of this work. *DESeq2,* indeed, provides a holistic solution for gene-based analysis of RNA-seq data: the use of shrinkage estimators significantly improves the stability and reproducibility of the analysis results, avoiding the problems related to the comparison of fold changes in RNA-Seq experiments. In addition, the *rlog* transformation, used for the implementation of shrinkage on fold changes, improves the visualization of expression levels, for example in heatmaps, and facilitates the application of other techniques such as Principal Component Analysis and clustering. The typical approach in searching for overexpressed genes among different experimental conditions consists in testing the null hypothesis that the logarithmic fold change (LFC) for a certain gene in one of the aforementioned comparisons is exactly zero: this means that the expression level of that gene is not affected by the status change, for example from healthy to severe form of the disease. One of the outputs of differential expression analysis is a list of overexpressed genes ranked by *p* value, representing the most statistically significant genes out of the input gene set. *DESeq2* package applies generalized linear models (GLM) on each gene to perform differential expression analysis [44]. A generalized linear model is built up of a linear predictor

$$\eta_{i}=\beta_{0}+\beta_{1}x_{1}+\ldots +\beta_{p}x_{\mathrm{pi}}$$

Along with two functions:

• The *link* function describes how the mean, $E\left(Y_{i}\right)=\mu_{i}$, depends on the linear predictor
  $$g\left(\mu_{i}\right)=\eta_{i}$$
• The *variance* function describing how the variance, $\mathrm{var}\left(Y_{i}\right)$ depends on the mean

$$\mathrm{var}\left(Y_{i}\right)=\emptyset V(\mu )$$

The dispersion parameter ∅ is a constant.

*DESeq2* approach starts by creating a count matrix K where each row is a gene and each column is a sample so that any element of the matrix uniquely identifies a specific gene within a specific sample. K matrix is modeled by following a negative binomial distribution (the so-called gamma-Poisson distribution), and its mean μ and dispersion α are calculated. The negative binomial distribution, like the Poisson distribution, describes the likelihood of the appearance of integers greater than or equal to 0. For this type of distribution, no equivalence between mean and variance is expected, suggesting that they can be easily used for approximating all those models in which variance and mean are different. The variance of a negative binomial distribution depends on its mean and on an extra parameter as well, called the dispersion parameter: the greater the dispersion, the more the variance tends to converge towards the same values, such as the mean, leading the negative binomial distribution to increasingly resemble a Poisson distribution. *DESeq2* works on the raw counts matrix, using normalization factors to limit the differences in library depth, and estimates the gene-wise dispersion to fit the negative binomial model. The normalization method used is DESeq2′s median of ratios: basically, all counts are divided by sample-specific size factors determined by the median ratio of gene counts relative to geometric mean per gene; the factors taken into account are sequencing depth and RNA composition. This kind of normalization is commonly used for gene count comparisons between samples and for differential expression analysis [45].

Finally, Wald or Likelihood Ratio tests are performed to test the null hypothesis. Wald test (also known as the Wald Chi-Squared test) is used to check the significance of certain parameters in terms of contributions to the model: if their values are zero, they can be removed from the model. Otherwise, they need to be included. A likelihood ratio test is performed to choose between two models by comparing their log likelihoods: if this difference is statistically significant, then the less restrictive model (the one with more variables) is chosen as the one which fits the data significantly better than, the more restrictive model. In this study, we decided to apply the Wald test to our data because, with equal statistical power, the Wald test is more suitable for the purposes of this work: the Likelihood Ratio test is usually preferred in the analysis of time course experiments and its high computational cost makes it impractical to apply. The *DESeq2* differential expression analysis produces a list of all differentially expressed genes along with log2 fold changes, p values and adjusted p values, which we will perform ontologies and gene set enrichment analysis upon.

### 2.4 Gene Enrichment Analysis

Gene Set Enrichment Analysis (GSEA) is an analytical method aimed at highlighting groups of genes that result over-represented between two biological conditions within a larger set of genes and inferring possible associations between those genes and different disease phenotypes (https://www.gsea-msigdb.org/gsea/index.jsp). One of the main tools used to perform GSEA is Gene Ontologies (GO): basically, GO is just like a structured vocabulary of biological terms, called GO terms, indeed. GO terms are subdivided into three macro-categories: Molecular Function (MF), Biological Process (BP), and Cellular Component (CC), and each of these terms are connected to a specific gene or protein through annotations based on literature. We used the *DOSE* R package [46] to perform enrichment analysis on our data. This package allows researchers to explore the level of similarity of diseases and gene functions through the Disease Ontology (DO) database, containing the descriptions of human genes involved in reported diseases. *DOSE* produces a large set of highly customizable figures, which make the results of the analysis even more readable, and which can be found in Supplementary Material. Also, the *clusterProfiler* R package (https://bioconductor.org/packages/release/bioc/html/clusterProfiler.html) was used to visualize enrichment results through a series of plots.

## 3 Results

### 3.1 Exploratory analysis results

**Fig. 1:**
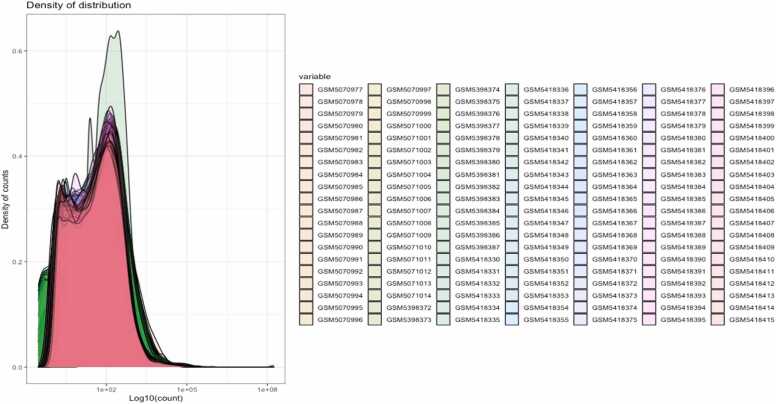
Density of counts taking into account the three datasets GSE178824 (16 samples from GSM5398372 to GSM5398387), GSE166424 (38 samples from GSM5070977 to GSM5071014) and GSE179448 (86 samples from GSM5418330 to GSM5418415).

**Fig. 2:**
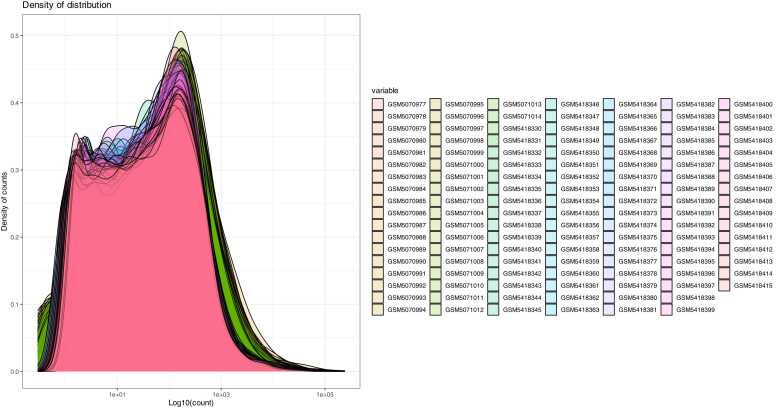
After removing dataset GSE178824, the distribution of normalized genes appears similar across all samples in the datasets.

Density plots show distributions of a continuous variable over a continuous or time interval. They make use of kernel density estimation to display the probability density function of the variable under examination. A kernel is defined as a special type of probability density function (PDF) with specific properties, for example it must be even, non-negative and real-valued [39]. Kernel density estimation is a non-parametric method for the estimation of the probability density function (PDF) of a continuous random variable. Non-parametric means that it does not assume any underlying distribution for the variable. The peaks show the points where the values of a variable are more concentrated in the defined range. Density plots are used to assess the quality of data by looking at the peaks in the plot: they can indicate that certain samples have a higher (or lower) number of reads mapped for that samples than the other samples. Fig. 1, Fig. 2 show the distribution densities of the variables relative to the mean of the normalized counts. The density plot should be similar across samples both in shapes and positions: if this does not happen, it is usually necessary to perform a within samples normalization [47]. The density plot of the distribution of normalized gene counts across all three datasets showed peaks in shapes related only to samples belonging to dataset GSE178824 (16 samples from GSM5398372 to GSM5398387), as shown in Fig. 1. We decided to completely remove this dataset as it could affect the results of the whole analysis. After removing dataset GSE178824, the density plot shows that the other two datasets, GSE166424 (38 samples from GSM5070977 to GSM5071014) and GSE179448 (86 samples from GSM5418330 to GSM5418415), have similar distribution both in shape and position (Fig. 2).

**Fig. 3:**
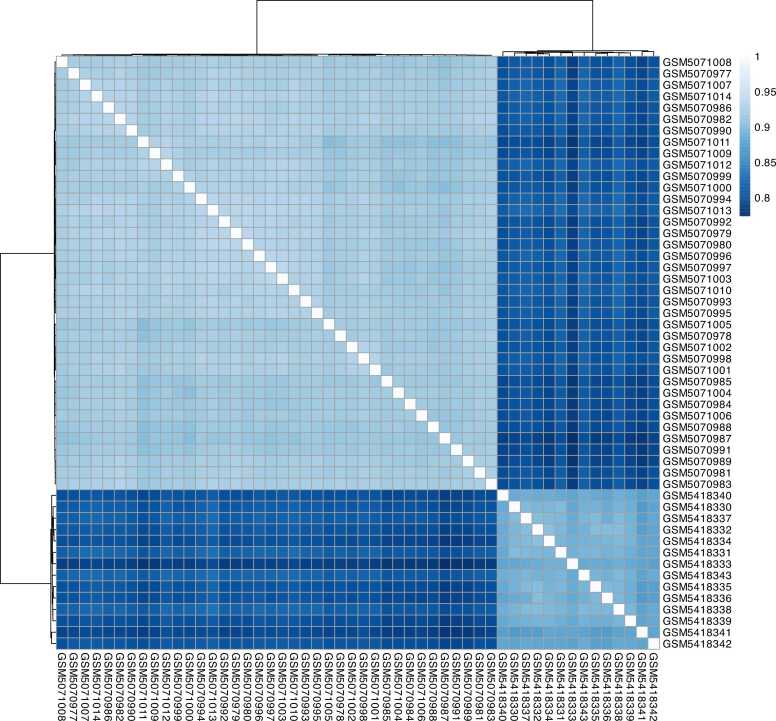
Spearman sample –to- sample correlation heatmap.

One of the most important preliminary steps in an RNA-Seq analysis is to assess the overall similarity between samples, expressed as the distance between each pair of samples calculated over the sample distance matrix. As a correlation paradigm, we choose Spearman correlation, which is a non-parametric algorithm that compares two ranked variables of either ordinal, interval, or ratio type and describes how well they can be described using a monotonic function. Unlikely parametric RNA-Seq can allow monitoring of the expression levels of a particular gene algorithm, no further assumptions on the data set are necessary to proceed with the similarity assessment. Spearman correlation evaluates monotonic relationships (linear or non-linear): perfect Spearman correlation of + 1 or − 1 only occurs when each of the variables is a perfect monotone function of the other, as shown in Fig. 3.

**Fig. 4:**
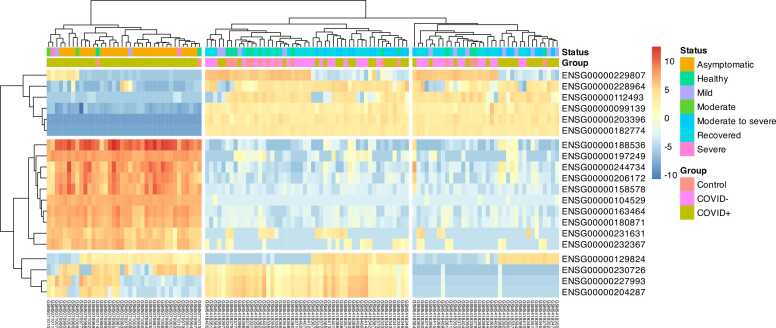
Top 20 most variable genes across samples.

Another way to examine the relationship among samples is using hierarchical clustering combined with heatmap, as shown in Fig. 4. We estimated the variance for each row in the log counts matrix of Euclidean distances from the logarithmic Counts Per Million (CPM), plotting the top 20 most variable genes across all samples. Row Z-Score displayed on the color key represents the scaling method used specifically for visualization in heatmaps, enhancing clusters of genes with similar trends in expression between different samples. Z-score is usually computed after the clustering so that it can only affect the graphical aesthetics and improve the color visualization. In this heatmap, raw Z-scores are calculated for each gene in each row and then plotted instead of the normalized expression values to ensure that the expression trends we wish to visualize are not exceeded by the expression values. Fig. 4 shows different clearly distinct clusters of genes, in which it is possible to notice the trend of the expression values of each gene with respect to each of the 124 samples to which they belong. The presence of these distinct rectangular zones indicates that those groups of genes (rows) are correlated with the corresponding groups of samples (columns). The color bar on the top of the plot indicates the group each gene belongs to: the clusters on the plot suggest that, at least apparently, the correlation between the genes and the samples of each cluster of genes does not depend on the group of origin of the genes themselves since the distribution of the various groups within each cluster seems to be completely random and has no clear distinctions.

**Fig. 5:**
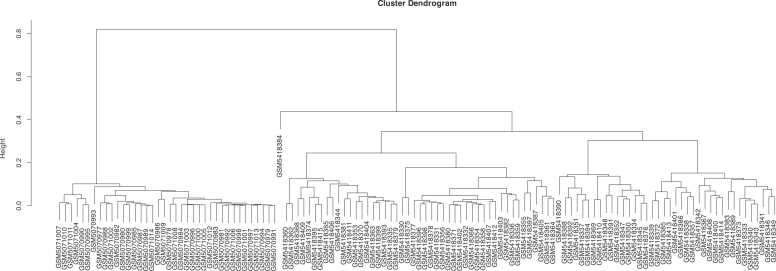
Dendrogram of hierarchical clustering of the log counts matrix of distances.

**Fig. 6:**
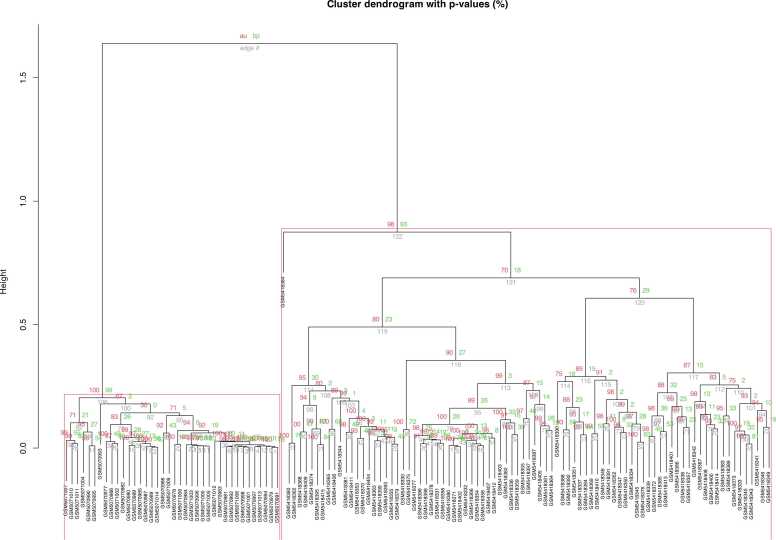
Results of bootstrapping applied to clustering. Red rectangles indicate the most stable clusters.

In order to assess the consistency of the obtained clusters, we decided to apply bootstrapping on them by using the *pvclust* R package [48]. After generating a high number of bootstrap samples by randomly sampling the element of the input data matrix, this algorithm is able to compute hierarchical clustering on each of them. Then, it evaluates each cluster measuring two parameters: the Bootstrap Probability (BP), which represents the frequency that one specific cluster is identified among the bootstrap copies, and the Approximately Unbiased (AU), which is the p-value calculated by multiscale bootstrap resampling. To facilitate the interpretation of bootstrapping results, we isolated the dendrogram from the rest of the heatmap (Fig. 5) and then reported the AU and BP values on each of the identified clusters (Fig. 6). The values on the dendrogram are AU (red, left), BP (green, right) and cluster labels (gray, bottom). Clusters showing AU>= 95 % are highlighted by rectangles and can be considered strongly supported by data (Fig. 6).

**Fig. 7:**
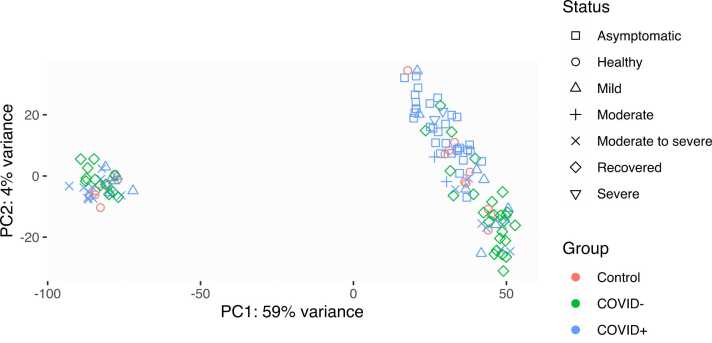
Principal Component Analysis. Data points represent the samples that are projected onto the 2D plane spreading along the two directions, which explains most of the differences between them.

Principal components analysis (PCA) is another method to visualize sample-to-sample distances. As shown in Fig. 7, the data points represent the samples, with different shapes and colors depending on the status or groups they belong to and are projected onto the 2D plane spreading along the two directions that explain most of the differences among them. The x-axis (PC1) is the direction that separates the data points the most. The y-axis (PC2) is a direction orthogonal to the first one that separates the data the second most. The percent of the total variance for each direction is printed on the axis label. Fig. 7 shows how samples having similar expression profiles are clustered together: we obtained two clusters of samples, which differ mainly by the group to which they belong rather than the status. Surprisingly, all asymptomatic samples (30 samples) are located only in the right cluster, along with the most part of recovered (25/37 samples) and all moderate (2 samples) and severe samples (2 samples). Mild (14 samples) and Moderate to severe (22 samples) samples are divided almost exactly into 2 clusters. Finally, most part of the Healthy control samples (10/17 samples) is in the right cluster. A similarity of the expression profiles can be traced back to a functional similarity between the various clusters, suggesting, in this case, that asymptomatic and recovered samples could have similar behavior from a genetic point of view.

**Fig. 8:**
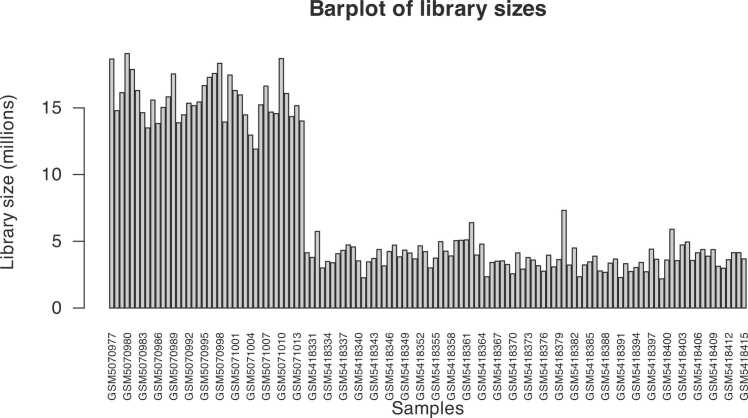
Barplot of library sizes.

**Fig. 9:**
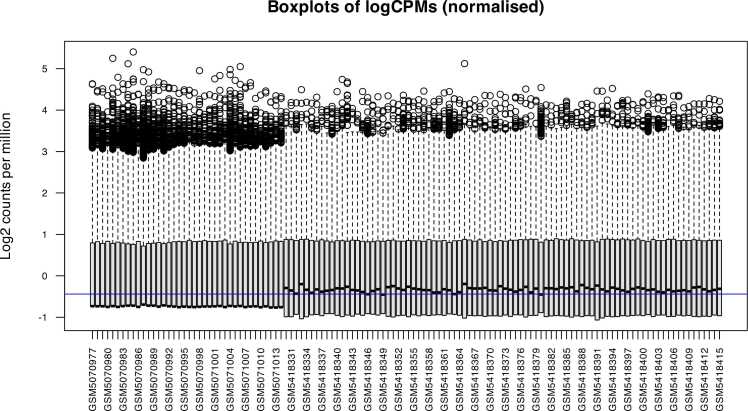
Boxplot of normalized logarithmic CPM.

Before proceeding with the differential expression analysis, it is usually necessary filtering out all those genes showing very low counts across all libraries because they would not provide enough evidence for differential expression and could interfere with some of the statistical approximations used in the pipeline. They also add to the burden of multiple tests when estimating false discovery rates, decreasing the ability to detect differentially expressed genes. In this study, we choose to keep genes only if they are expressed at a counts-per-million (CPM) above 0.5 in at least two samples. A general rule to choose a good threshold could be identifying the CPM that corresponds to a count of 10, which in this case is about 0.5. After removing all lowly expressed genes, it is possible to look at the quality of data plotting the library size as shown in Fig. 8: we can see that count data is not normally distributed, so we need to log the counts in order to examine the distributions of normalized counts and then use box plots to check the distribution of the read counts on the log2 scale, as shown in Fig. 9.

### 3.2 Differential expression analysis

**Fig. 10:**
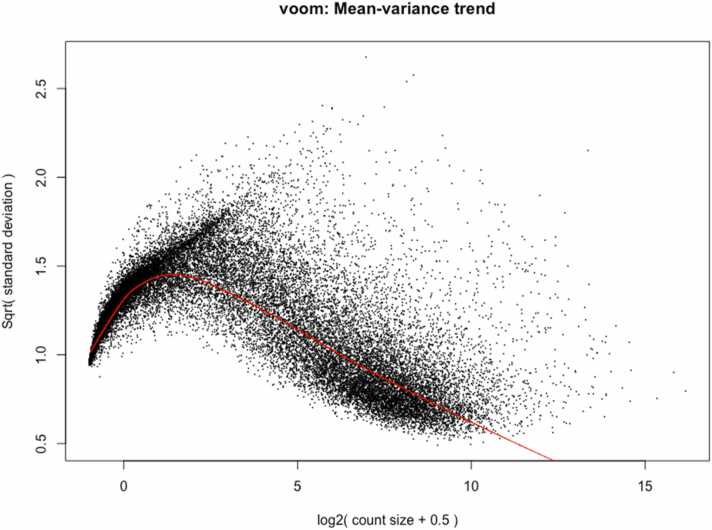
Mean – variance trend.

Variance Modelling at the Observational level (VOOM) method was used to estimate the mean-variance relationship to understand how the variability of RNA-Seq read counts depends on the size of the counts [49]. In Fig. 10, gene-wise square-root residual standard deviations are plotted against average log-count: gene-wise means and variances of RNA-Seq data are represented by black points. The mean-variance trend is represented by a red line and allows each observation to be mapped onto a square-root standard deviation value using its fitted value for log-count. Typically, this kind of plot shows a decreasing trend between the means and variances, as shown in Fig. 10.

The most important part of the entire workflow is the identification of all those genes showing any difference in their expression values in a particular experimental condition. This difference is assessed both in terms of fold-change (FC) and statistical significance (p-value). In this study, we focused on the following groups:

1. "Control_Healthy" vs "Recovered"
2. "Control_Healthy" vs "Asymptomatic"
3. "Control_Healthy" vs "Mild"
4. "Control_Healthy" vs "Moderate"
5. "Control_Healthy" vs "Moderate to severe"
6. "Control_Healthy" vs "Severe"
7. "Asymptomatic " vs "Severe"

For each comparison, a corresponding table showing the complete results of the differential expression analysis has been produced and can be found in Supplementary Material (https://github.com/COMBINE-Group/Identification-of-differentially-expressed-genes-as-potential-novel-COVID-19-biomarkers-through-comp.git)*,* along with the complete series of plots produced at the various stages of the analysis. Tables 1–7 (provided as supplementary material) show the most overexpressed genes (up and down-regulated) for each condition, where *base mean* is the mean of normalized counts of all samples, *log2FC* is the logarithm of the fold change, *lfcSE* is the standard error of the log2FoldChange estimate, the *stat* is Wald statistic, *pvalue is* Wald test p-value and *padj* is Benjamini-Hochberg adjusted p-values.

**Fig. 11:**
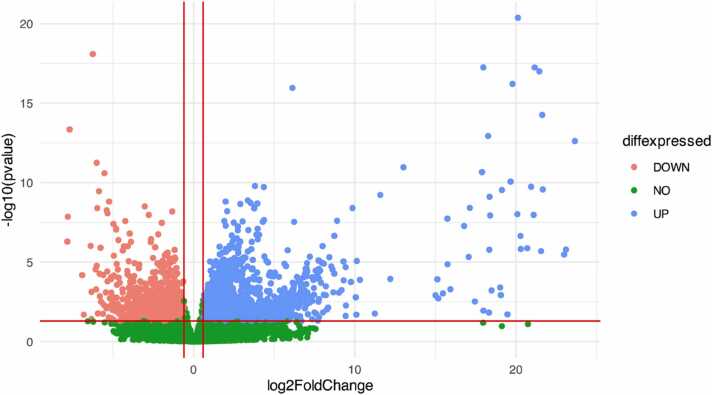
Volcano plot for “Asymptomatic vs Severe” condition.

Volcano plots are a very good method to give an overview of the expression level of all genes for a specific condition in a clear and intuitive way. Fig. 11 shows the Volcano plot for the “Asymptomatic vs Severe” condition. Among the up-regulated genes (blue points on the right side of this figure) in the "Asymptomatic" vs "Severe" contrast, we identified four particular genes which resulted in being up-regulated in Severe condition and not differentially expressed in the Asymptomatic one:

• ENSG00000077348 (EXOSC5)
• ENSG00000117899 (MESD)
• ENSG00000076043 (REXO2)
• ENSG00000099899 (TRMT2A)

We decided to focus on the differentially expressed genes for “Asymptomatic” and “Severe” conditions in the next steps of the analysis. Both these conditions represent two key points in the fight against COVID-19: asymptomatic patients represent the main vector of the spread of the virus because they are difficult to identify and, as a result, are not treated properly and in time, allowing the virus to circulate without almost any control. Severe patients, on the other hand, are those most at risk, are almost always hospitalized and, unfortunately, tend to have often permanent and serious consequences, even in cases where therapy is successful. In both cases, early diagnosis is decisive in terms of both control of the spread and treatment of the disease, and it is for this reason that this study has focused mainly on the analysis of differentially expressed genes for these two particular conditions.

### 3.3 Enrichment analysis results

**Fig. 12:**
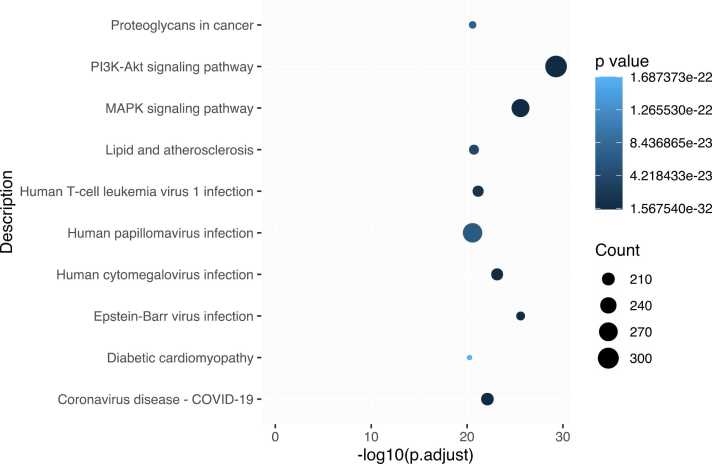
Top 10 GO terms plotted for condition “Asymptomatic vs Severe”.

*DOSE* R package (https://www.rdocumentation.org/packages/DOSE/versions/2.10.6) was used with the *clusterProfiler* R package (https://bioconductor.org/packages/release/bioc/html/clusterProfiler.html) to perform enrichment analysis in order to discover disease associations of high-throughput biological data. Both these packages produce different plots to show the results of the analysis. Fig. 12 shows the most enriched GO terms for the differentially expressed genes in condition "Asymptomatic " vs "Severe": the size of the point represents the number of differentially expressed genes for that GO term, and the color depends on the p value. Notably, the PI3K-Akt signaling pathway is a metabolic pathway involved in several biological processes, such as viral replication, which makes it a possible target in COVID-19 therapy [50]. Furthermore, the MAPK signaling pathway is involved in many cellular processes essential for immune system function: in particular, recent studies show that the p38 MAPK inhibitor reduces the gene expression levels of *TNF-α* and other inflammatory cytokines that were increased during the SARS-CoV-2 infection, thus making p38 MAPK a potential pharmacological target for COVID-19 [51].

**Fig. 13:**
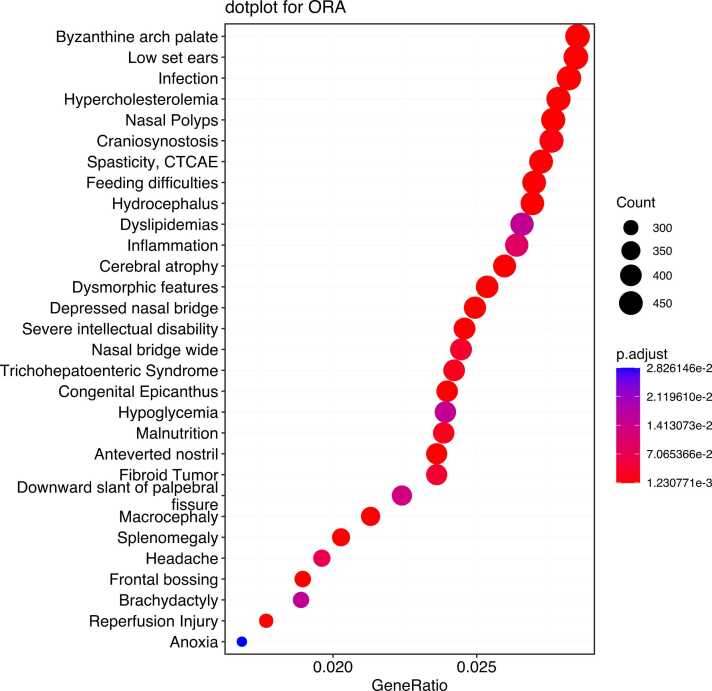
Dotplot of Over Representation Analysis results for “Asymptomatic vs Severe” condition.

**Fig. 14:**
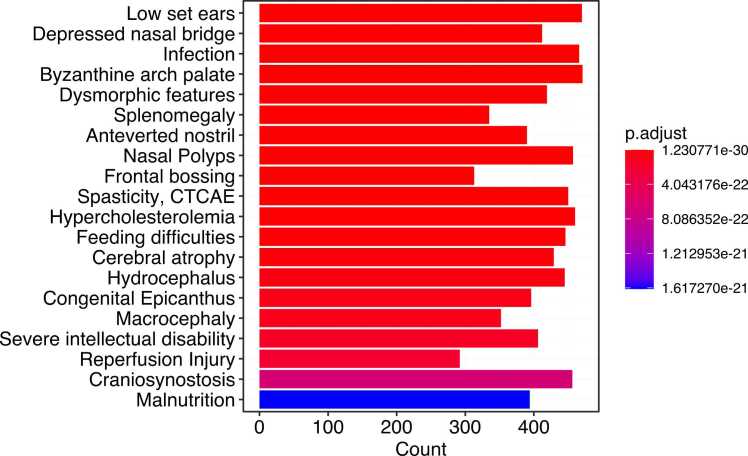
Barplot of Over Representation Analysis results for “Asymptomatic vs Severe” condition.

Over-representation analysis (ORA) has been performed to determine whether genes from pre-defined gene sets, for example, those genes belonging to specific GO terms or pathway, are presently more than would be expected (over-represented) in the subset of data under examination. The results of this analysis can be visualized both as a dotplot, as shown in Fig. 13, and as a barplot, as shown in Fig. 14. In both figures, the color depends on the p-value, and the size of the dot or the length of the bar varies with the gene counts. Interestingly, among the most significant pathways, Infection, Inflammation and Headaches, which are some of the symptoms of COVID-19, stand out.

## 4 Discussion

COVID-19 has a different range of clinical manifestations, on the basis of which COVID-19 patients are categorized as asymptomatic, mild, moderate, and severe. According to some data, 81 % of patients with COVID-19 had a mild or moderate condition, 14 % had severe disease, and 5 % had critical illness [52], [53]. Asymptomatic people are the primary vectors for distributing the virus in a population [54,48]. On the other hand, severe patients present the worst manifestations of the disease, especially in the lungs [55]. Numerous evidence demonstrates that the host immune response and human proteins from different cellular pathways to SARS-CoV-2 play an essential role in infection pathogenesis and clinical manifestations [56].

Researchers have been exploring different compounds and approaches for the treatment of COVID-19. One of the beneficial approaches in addition to an in vitro and in vivo investigation, is a molecular computational investigation, the in silico molecular docking. Using this approach, polyphenolics and 1,3,4-oxadiazoles have been identified as potent bioactive compounds with antiviral properties. Specifically, Taroxaz-104 shows highly potent inhibitory interactions with nCoV-RdRp-RNA, making it a promising candidate for targeting one of the potential active sites of nCoV-RdRp [57]. Cordycepin, a natural adenosine analogue, has also shown strong antiviral properties against SARS-CoV-2, with recent studies showing it can be more effective than remdesivir in inhibiting the multiplication of new resistant strains of the virus [58]. Additionally, both natural and synthetic nitrogenous heterocyclic antivirals have become popular choices for COVID-19 treatment, with three antioxidant nitrogenous heterocyclic compounds of the 1,3,4-thiadiazole type identified as effective inhibitors of SARS-CoV-2 replication. These compounds, CoViTris2022, Taroxaz-26, and ChloViD2022, showed lower anti-RdRp and anti-SARS-CoV-2 EC50 values than molnupiravir [59]. Finally, didanosine (DDI), a synthetic compound used to treat HIV/AIDS, has shown potential as an effective anti-COVID-19 drug, with tests on new virulent strains of SARS-CoV-2 using an in silico molecular docking interpretation and biological evaluation [60].

In our research, we selected three gene expression profiles (GSE166424, GSE178824, and GSE179448) related to SARS-CoV-2 from NCBI GEO datasets. We focused on genes that were up-regulated in patients with severe COVID-19 condition, in comparison to other groups. We identified four up-regulated genes in the severe condition: TRMT2A, EXOSC5, REXO2, and MESD. The same four genes were noticed to be non-differentially expressed in the asymptomatic condition, and only one of these, TRMT2A, was up-regulated in the moderate one. On the other hand, these four genes are not present in the moderate to severe, mild, and recovered conditions. TRMT2A gene encodes a protein characterized as a biomarker of increased risk of recurrence in HER2 + breast cancer patients [61]. According to certain clinical and genetic studies, EXCOSC5 gene is found to be related to a disease spectrum that includes also cardiac conduction abnormalities and arrhythmias [62]. EXCOSC5 was also recently shown to underlie an autosomal recessive neurodevelopmental condition characterized by hypotonia, delay in development, dysmorphic facies, and anomalies in the cerebellum [62]. REXO2 is a gene encoding a protein involved in DNA replication, repair, recombination, and RNA processing and degradation. It also has a remarkable function in the resistance of human cells to UV-C-induced cell death through its significant function in the DNA repair approach [63]. MESD is a critical gene for specifying embryonic polarity and mesoderm infusion. Recently, mutations impacting MESD, encoded for a chaperone demanded the lipoprotein receptors LRP5 and LRP6 in the endoplasmic reticulum, have been demonstrated to be the cause of autosomal recessive OI XX in homozygous youths [64]. The up-regulation of these four genes in people with severe COVID-19 disease, the fact that they are not differentially expressed in asymptomatic people, as well as their absence in people with mild, moderate, and recovered conditions, may indicate that the expression of these genes in the host could affect the degree of disease severity.

Moreover, we focused on the most overexpressed genes in severe COVID-19 patients. Our results have shown that POLR2A gene is overexpressed and up-regulated in patients with severe COVID-19 condition. This gene encodes a protein which has a crucial interaction with the virus polymerase, thus having a key role in the viral replication and in its transcriptional activity [65]. The role of that gene in the virus activity may explain its up-regulation in severe COVID-19 patients.

APOBEC3A (also called A3A) is one of the most down-regulated genes in patients with severe COVID-19 condition. Generally, the APOBEC3 (A3) family has a key role in the immune defence mechanism against viral infections by producing deadly mutations in viral genomic DNA or RNA in infected cells. In particular, A3A is significantly induced after INF-gamma stimulation [66]. The role of A3A may explain why in our results it is down-regulated in severe COVID-19 patients, who may present a lack of their immune response. Interestingly, we also noticed that the A3A protein follows an up-regulation pattern in asymptomatic patients, and this may be related to their good immune response.

HLA-DQA1 gene, which encodes the HLA class II histocompatibility antigen, DQ alpha 1 chain, appears also to be down-regulated in severe COVID-19 patients. Several studies have investigated the role of human leukocyte antigen (HLA) genes in COVID-19 disease and their association with the severity of the disease [67], [68], concluding that the different genetic structure of the immune system across many regions and ethnic groups must be taken into account, probably being the association between COVID-19 ethnic-dependent [68].

We also investigated the most overexpressed genes in the asymptomatic condition, focusing on the most up-regulated gene, as well as the most down-regulated one. TNF gene is the most up-regulated gene in the asymptomatic condition, and it is one of the genes that encode the tumour necrosis factor (TNF). TNF acts both as an adipokine and a cytokine; in the latter case, it is released by macrophages, as part of an inflammatory response, to alert the other immune cells. In our results, such as most of the literature data suggest, it is shown to be up-regulated also in the severe condition, but its p-value is dramatically lower in the asymptomatic condition compared to the severe one. This means that, in our study, this gene is more up-regulated in patients with asymptomatic COVID-19 disease, than in patients with a severe condition. Generally, an increased tumour necrosis factor α (TNF-α) characterizes a severity of the disease [69]. Therefore, our results may differ from literature data because of some pre-existing patient conditions among our asymptomatic samples, such as comorbidities or other inflammatory diseases.

The most down-regulated gene in the asymptomatic condition is the KIR2DS4 gene, which encodes the human killer cell immunoglobulin-like receptor 2DS4 protein. Killer cell immunoglobulin-like receptors (KIRs) are expressed by natural killer cells (NKcs) and specific T cell subsets. In the work of Bernal et al. [70], the results indicate that NKcs play a significant role in the clinical diversity of COVID-19, with certain KIR/ligand interactions related to disease severity. The KIR2DS4 gene down-regulation we found in asymptomatic patients is in line with the results about the KIRs’ role in the severity of COVID-19 disease, because the down-regulation of this gene in asymptomatic patients means that, in those people, the protein is less expressed.

The pathogenesis of SARS-COV-2 infection may vary in different patients, and the genetic component of the subject certainly plays an important role. Finding genes that could be involved in the different grades of severity of the COVID-19 disease can be a useful tool in clinical practice. Being able to understand which genes are mostly involved in a severe condition of the disease could be fundamental in predicting the most harmful symptoms and to act promptly on the patient in this context.

In a similar investigation, genome association studies using RNA-Seq data were conducted to identify highly expressed gene biomarkers for COVID-19 and develop a machine learning model for analysis. Through a combination of bioinformatics strategies, statistical techniques, and machine learning algorithms, potential biomarkers were extracted and analyzed, identifying 67 genes as promising biomarkers comprising 49 up-regulated and 18 down-regulated genes. Among the classifiers scrutinized, the Support Vector Machine (SVM) exhibited the highest accuracy among the classifiers tested, highlighting its potential for predicting COVID-19 based on the identified differentially expressed genes [71].

Understanding the biology and functional significance of the four obtained genes (TRMT2A, EXOSC5, REXO2, and MESD) provides insights into the molecular processes associated with disease severity in COVID-19. Integrating these potential biomarkers into clinical practice may hold several potential implications. Firstly, they can aid in risk stratification and early identification of patients at higher risk of developing severe symptoms. This knowledge can guide allocation of healthcare resources and intensify monitoring or treatment interventions for high-risk individuals. Secondly, these candidate biomarkers can be incorporated into diagnostic tests to improve accuracy in predicting disease outcomes. Thirdly, targeted therapies can be developed to modulate the pathways associated with these biomarkers, aiming to mitigate disease severity and improve patient outcomes. However, further validation studies are essential to establish the robustness and generalizability of these potential biomarker genes across diverse populations. Additionally, exploring their utility in combination with other established biomarkers or clinical indicators may enhance predictive accuracy and clinical decision-making.

## 5 Conclusion

The pathophysiology and severity of COVID-19 differ from patient to patient and depend partly on underlying chronic disease and risk factors. Our research presents specific hypotheses that connect host genetic diversity to symptom profiles and laboratory characteristics. RNA-Seq data analysis for transcript identification and the quantification of gene expression represents a powerful tool that finds a wide variety of applications; in this study, performing differential expression analysis on RNA-Seq data allowed us to detect and highlight all those genes involved in COVID-19 progression whose expression level may vary changing from a specific grade of disease severity to another, helping us to go deeper into the genetic causes of COVID-19. We specifically designed and analyzed 7 different contrasts to investigate all possible biological conditions related to specific disease states and infer consistent conclusions about the expression level of particular groups of genes in different stages of the disease. Our analysis produced a list of differentially expressed genes (DEGs) for each condition, upon which we also performed a Gene Set Enrichment Analysis (GSEA), to highlight any possible correlation between genes in our lists and different disease phenotypes in which our genes resulted enriched. The identification of genes that turn out to be overexpressed in a specific grade of disease severity, could help to think about them as potential biomarkers for that grade of disease severity.

In particular, we focused on severe and asymptomatic conditions and performed an accurate literature search on some of the most up- and down-regulated genes in the correspondent DEGs list, which we found particularly interesting. We identified four genes being up-regulated in the severe condition and not-differentially expressed in the asymptomatic condition: TRMT2A, EXOSC5, REXO2, and MESD. Moreover, we found out that POLR2A, a gene playing a crucial role in virus polymerase, is up-regulated in severe conditions. Finally, we identified APOBEC3A as the most down-regulated gene in asymptomatic conditions: APOBEC3A family is strongly involved in immune defense mechanisms against viral infection.

For future study, a more detailed investigation of the identified genes may provide a context to introduce new human gene biomarkers for different conditions of COVID-19 disease for rapid identification and treatment. In particular, having regard to the connection of many of the identified genes with other diseases, such as various types of cancer, a possible application of the obtained results could be a thorough analysis of the effects of COVID-19 infection on patients already suffering from particular types of cancer, or conversely, how the progression of a tumor can change on COVID-19 patients. Moreover, these differentially expressed genes can be effectively used in profiling digital patients for their usage in modelling and simulation platforms dealing with COVID-19. In this respect, among the potential future developments for this study, we envisage the combined use of the Universal Immune System Simulator for SARS-CoV-2 (UISS-COVID-19 for short). UISS-COVID-19 is an in silico trial platform able to simulate and predict the intricate human immune system dynamics in response to SARS-CoV-2 insult [72], [73]. Mapping the cellular and molecular effects of these genes into the UISS-COVID-19 platform can contribute to tailor predictions to achieve more precise individualized simulations.

Undoubtedly, the results obtained through this in silico pipeline would require further confirmation studies to assess the credibility of the selected potential biomarkers; however, the in silico predictions could be used as input for the designing and refinement of new clinical trials.

The RNA-Seq methodology, while widely used in research, is still relatively young and continuously evolving. The ease in the application and interpretation of the results are certainly one of its strengths and among the main reasons why it is so widespread. RNA-Seq computational pipelines are many and all make use of different computer tools. For this reason, there is still no universally recognized golden standard that would increase the credibility and reliability of such techniques, as well as help less experienced users. The absence of a standard imposes a great effort on the scientific community: who is going to use for the first time an RNA-Seq pipeline has to choose between thousands of different tools, not always properly explained, and the application of the various stages that constitute the pipeline, some of which are absolutely necessary, is not immediately understood. Many studies, such as [74], focus on the evaluation of the performance of the currently available tools for differential expression analysis of RNA-Seq data for the purpose of providing guidelines for users: *DESeq2* R package has been recognized as one of the most reliable and accredited tools among all those tested.

In this work we presented a complete RNA-Seq pipeline which, starting from publicly available datasets and using specific tools such as *DESeq2* R package, leads to the identification of potential biomarkers through a long series of steps that we have tried to explain in as much detail as possible. We trust that, beyond the potential discovery of the candidate biomarkers presented, which represent the main objective of this work, it can also be of help to those who approach this type of analysis for the first time.

## CRediT authorship contribution statement

**Valentina Di Salvatore:** Writing – original draft, Software, Methodology, Writing – review & editing. **Avisa Maleki:** Data curation, Writing – review & editing. **Elena Crispino:** Data curation, Writing – review & editing. **Giulia Nicotra:** Writing – review & editing. **Giulia Russo:** Writing – review & editing, Formal analysis. **Francesco Pappalardo:** Conceptualization, Supervision, Writing – original draft.

## Declaration of Competing Interest

The authors declare no conflict of interest.
