# Supplementary material for: Computational identification of differentially-expressed genes as suggested novel COVID-19 biomarkers: A bioinformatics analysis of expression profiles
Source: Comput Struct Biotechnol J. 2023 Jun 12;21:3339–54. doi: 10.1016/j.csbj.2023.06.007 (PMC10259169; doi:10.1016/j.csbj.2023.06.007)
Supplement: Supplementary file 1 — Supplementary material [file mmc1.zip › mmc1/Plots.zip!/Plots/Res4_plots/GOTerms_Res4.pdf]

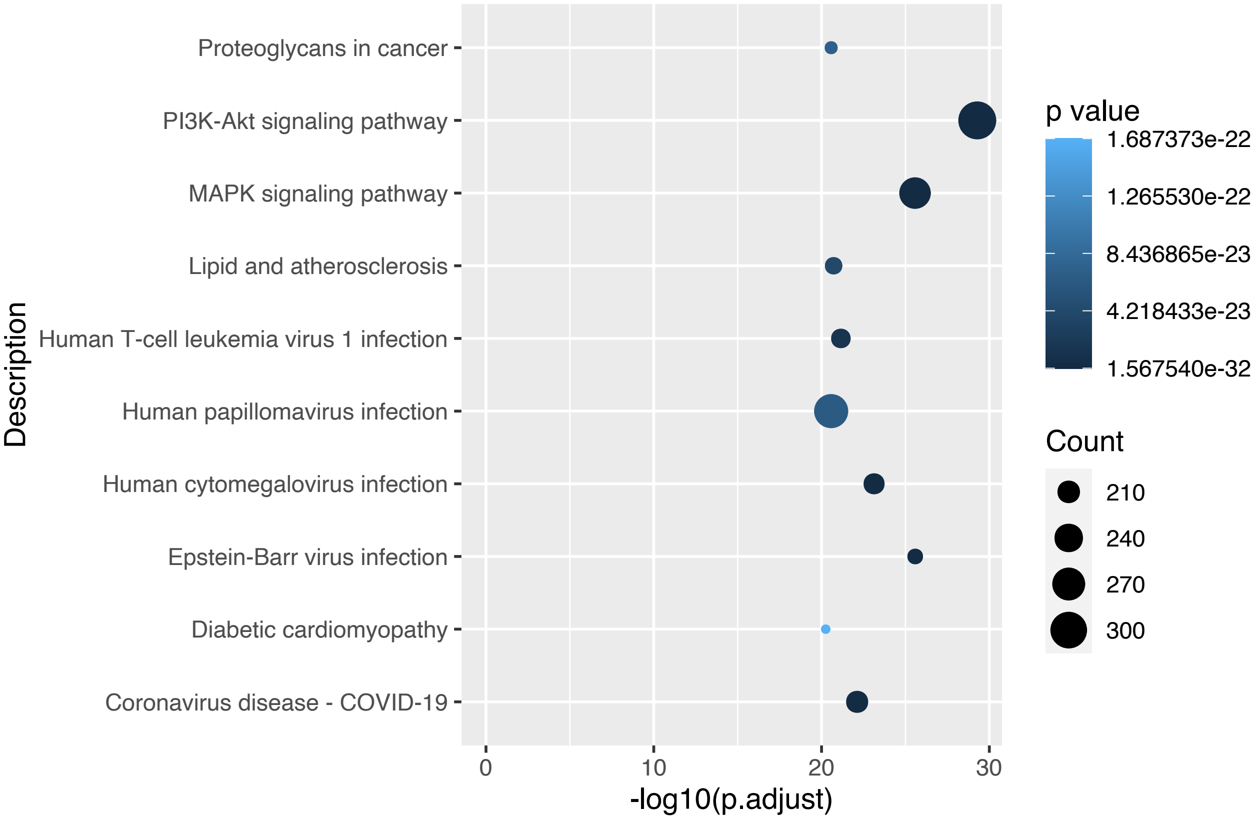
Proteoglycans in cancer
PI3K-Akt signaling pathway
MAPK signaling pathway
Lipid and atherosclerosis
Human T-cell leukemia virus 1 infection
Human papillomavirus infection
Human cytomegalovirus infection
Epstein-Barr virus infection
Diabetic cardiomyopathy
Coronavirus disease - COVID-19
Description
0
10
20
30
-log10(p.adjust)
p value
1.687373e-22
1.265530e-22
8.436865e-23
4.218433e-23
1.567540e-32
Count
210
240
270
300
